# Practical Notes on Diagnosis and Treatment

**Published:** 1908-10-17

**Authors:** 


### Practical Notes on Diagnosis and Treatment.

**Dermatitis.**

An acute eczema, or other form of acute dermatitis, of limited distribution, finds a suitable application in the following ointment: Zinc ointment, vaseline, of each 1 oz., liniment of lime, ½ oz. The ointment should be applied thickly spread on lint. Before a fresh application is made the affected part should be wiped with a soft towel. It should not be washed.

**Pleurisy in Childhood.**

In young children and infants pleurisy is certainly a puzzling illness. The secret of this lies principally in the detrimental effects of *pain* in such patients. Pain may kill an infant from the shock and suffering it entails, and yet the widespread fear of opium in the illnesses of childhood may lead to very imperfect attempts for its relief.—*Dr. Poynton.*

**Venesection in High Blood Pressure.**

In cases of very young men with very high and obstinate blood pressures, I think that venesection has an indubitably good effect. I have had the most grateful letters from hyperpietic patients telling me that for years they had not felt so well as for some months after each time they had been bled.—*Professor Clifford Allbutt.*

**Gastric Tetany.**

It is a mistake to hold, as physicians mostly do, that gastric tetany is almost necessarily fatal. Under efficient surgical treatment hardly any case is hopeless.—*Mr. Mayo Robson.*

